# Effectiveness of Platelet-Rich Plasma for Patients With Carpal Tunnel Syndrome: A Systematic Review and meta-Analysis of Current Evidence in Randomized Controlled Trials

**DOI:** 10.3389/fphar.2022.834213

**Published:** 2022-04-27

**Authors:** Jiabao Jiang, Fei Xing, Rong Luo, Ming Liu

**Affiliations:** Department of Orthopedics, Orthopedic Research Institute, West China Hospital, Sichuan University, Chengdu, China

**Keywords:** carpal tunnel syndrome, local injection, systematic review, steroid, platelet-rich plasma

## Abstract

**Background:** Recently, there was a series of clinical studies focusing on local injection of platelet-rich plasma (PRP) for treatment of patients with carpal tunnel syndrome (CTS). However, the safety and efficacy of PRP in these CTS patients remains controversial. Therefore, we performed a systematic review to compare PRP with other conservative treatments in treatment of CTS patients.

**Methods:** We systematically searched from electronic databases (Cochrane, PubMed, Web of Science, and EMBASE) up to 10 December 2021. The data of clinical results were extracted and analyzed by RevMan Manager 5.4.

**Results:** Finally, eight randomized controlled studies, involving 220 CTS patients undergoing local injection of PRP were enrolled in this systematic review. All enrolled trials were considered to be of high quality. In the short-term efficacy, the PRP group was significantly lower in symptom severity scale (SSS) compared with the control group (MD = −2.00; 95% CI, −3.15 to −0.85; *p* = 0.0007; I^2^ = 0%). In the mid-term efficacy, the PRP group was significantly effective than the control group in the visual analogue scale (MD = −0.63; 95% CI, −1.22 to −0.04; *p* = 0.04; I^2^ = 61%), SSS (MD = −3.56; 95% CI, −4.93 to −2.18; *p* < 0.00001; I^2^ = 0%), functional status scale (MD = −2.29; 95% CI, −3.03 to −1.56; *p* < 0.00001; I^2^ = 45%), sensory peak latency (MD = −0.39; 95% CI, −0.58 to −0.19; *p* = 0.0001; I^2^ = 0%) and cross-sectional area of median nerve (MD = -0.20; 95% CI, −0.31 to −0.10; *p* = 0.0002; I^2^ = 0%). In the mid-long-term efficacy, the PRP group was only significantly lower in SSS compared with the control group (MD = −2.71; 95% CI, −4.33 to −1.10; *p* = 0.001; I^2^ = 38%).

**Conclusion:** Local PRP injection is more effective than other conservative treatments in terms of mid-term efficacy in relieving pain, improving wrist function and symptoms, reducing MN swelling, and partially improving electrophysiological indicators. However, the long-term adverse side and consensus on standardization of PRP in CTS patients still need further large-scale trials.

## Introduction

As a compressive peripheral nerve disease, carpal tunnel syndrome (CTS) is caused by the median nerve (MN)’s compression by increased pressure in the bone fiber tunnel formed by wrist bones, transverse carpal ligament, and digital flexor tendons (Chammas, 2014). The clinical symptom of CTS includes pain, numbness, weakness, and paresthesia in the three and a half fingers on the radial side. What’s more, as the disease progresses, muscle atrophy in the thenar area and decreased hand muscle strength will occur (Alfonso et al., 2010). The previous studies reported that the annual incidence of CTS was estimated to be 90 new cases per 100,000 men and 193–280 new cases per 100,000 women (Latinovic et al., 2006; Bongers et al., 2007).

Currently, various conservative treatments, including wrist splinting, local steroid injections, oral medications, and acupuncture are used for mild to moderate CTS. However, the results of these conservative treatments are not satisfactory to patients (Ostergaard et al., 2020). The previous study showed that about half of the CTS patients treated conservatively finally received surgery (Burton et al., 2016). Platelet-rich plasma (PRP) is a concentrated product of autologous blood, containing concentrated platelets and various growth factors (Razmara et al., 2008; Ruzafa et al., 2021a; Zhou et al., 2021). PRP not only has a good ability of anti-inflammatory and tissue repair, but also can promote the regeneration of peripheral neurons (Zheng et al., 2016; Ruzafa et al., 2021b; Negrini et al., 2021). PRP has been widely used in refractory wounds, external humeral epicondylitis, and plantar fasciitis (Chen et al., 2021a; Fei et al., 2021; Zhang et al., 2021). Recently, the application of local PRP injection has gradually applied in the treatment of CTS. Compared with other conservative managements, the clinical effect of local injection of PRP in the treatment of CTS patients is still controversial.

Therefore, we performed this systematic review and meta-analysis to evaluate the efficacy and safety of local PRP injection in treatment for CTS. The study will be evaluated from the following aspects: the visual analogue scale (VAS), Boston carpal tunnel questionnaire (BCTQ), cross-sectional area (CSA) of MN and electrodiagnostic examination parameters including distal motor latency (DML), sensory nerve conduction velocity (SNCV), sensory peak latency (SPL). BCTQ is the most commonly used self-assessment questionnaire for CTS patients, including two subscales, symptom severity scale (SSS) (11 items) and functional status scale (FSS) (8 items). The score of each item ranges from 0 to 5. The higher the score, the higher the severity and disability (Levine et al., 1993).

## Methods

The Preferred Reporting Items for Systematic reviews and Meta-Analysis (PRISMA) guidelines and Quality of Reporting of Meta-analyses (QUORUM) guidelines were followed in this systematic review. In our study, we created a prospective protocol, consisting of objectives, study selection strategies, inclusion criteria, exclusion criteria, statistical analysis, and outcome measures.

### Search Strategy

Two reviewers independently searched for potentially relevant published and unpublished studies using electronic databases, including Cochrane, PubMed, Web of Science, and EMBASE, from inception to 10 December 2021. The following keywords were used for the search: “Carpal Tunnel Syndrome,” “Carpal Tunnel Syndromes,” “Syndrome, Carpal Tunnel,” “Syndromes, Carpal Tunnel,” “Amyotrophy, Thenar, Of Carpal Origin,” “Median Neuropathy, Carpal Tunnel,” “Compression Neuropathy, Carpal Tunnel,” “Entrapment Neuropathy, Carpal Tunnel,” “Platelet-Rich Plasma,” “Plasma, Platelet-Rich,” “Platelet Rich Plasma,” “PRP”. After the electronic search is completed, manual searches were carried out on related literatures and references to find potential eligible studies.

### Eligibility Criteria

The inclusion criteria for this study were as follows: 1) Population: patients were adults and diagnosed with CTS. 2) Intervention: patients were treated with local PRP injection. 3) Comparator: patients who were treated with other conservative management, such as wrist splint and local injection with methylprednisolone acetate, triamcinolone, hyaluronidase, dextrose and normal saline. 4) Outcomes: one of the following results was reported, VAS, BCTQ including SSS and FSS, CSA of MN and electrodiagnostic examination parameters including DML, SNCV, SPL. 5) Study design: The studies were original, randomized control trials (RCTs) only. 6) The studies report PRP’s preparations and injection procedures performed in CTS patients.

The exclusion criteria for this study were as follows: 1) Studies not published in English 2) Retrospective studies and Cohort studies. 3) Animal studies. 4) Nonoriginal research, such as reviews, technical reports. 5) Duplicated publications. 6) Single abstracts 7) Case reports. 8) Studies in which the relevant data cannot be extracted and the original author is contacted without a response. If there is a dispute between the two reviewers, it will be settled through consultation with a third reviewer.

### Data Extraction

Two reviewers scanned all enrolled studies to extract data independently according to the inclusion and exclusion criteria. The demographic characteristics extracted for meta-analysis were as follows: first author, publication year, country, number of patients in different groups, male/female ratio, the average age of patients, duration of symptoms, grade of CTS, details of the comparator, outcome measures, and duration of follow-up. And extraction of related complications after local injection. In enrolled trials with more than two control groups, we only extracted the data from PRP and other conservative groups. In this study, we define the outcomes around 1 month after the intervention as short-term efficacy, 3 months as mid-term efficacy, and 6 months as mid-long-term efficacy. All data were entered into an electronic spreadsheet. Furthermore, any disagreements were resolved by discussion and consensus with a third reviewer.

### Assessment of Methodological Quality

The methodological quality of trials included in this study was evaluated independently by two reviewers, according to Cochrane Collaboration for Systematic Reviews (Corbett et al., 2014). The following items were considered: random sequence generation, allocation sequence concealment, blinding of participants and personnel, blinding of outcomes assessment, incomplete outcome data, selective reporting, and other bias. Each item was assessed as “Low risk of bias,” “Unclear risk of bias,” or “High risk of bias.” If the item was reported incorrectly, the judgment was “High risk of bias.” If the item was reported inadequately, the judgment was “Unclear risk of bias.” If the item was reported correctly and adequately, the judgment was “Low risk of bias.” Any disagreements were resolved by discussion and consensus with a third reviewer.

### Statistical Analysis

The statistical analysis was independently performed with RevMan software (Version 5.4; Copenhagen: The Nordic Cochrane Centre, The Cochrane Collaboration, 2020) by two reviewers. The mean difference (MD) between groups of PRP and control were reported with 95% confidence interval (95% CI) and performed to evaluate continuous variables. To measure heterogeneity between studies, we used the I^2^ statistic. Furthermore, heterogeneity was accepted, and the randomized-effects model was performed, when I^2^ was>50%. Otherwise, the fixed-effects model was performed. Forest plots were used to graphically represent the difference in outcomes of groups of PRP and control and for all included studies. If *p* values were <0.05, the results were considered statistically significant. We did not conduct a publication bias assessment because when the number of studies included in the meta-analysis is less than 10, it is unnecessary to conduct a publication bias assessment (GS HJ., 2011).

## Results

### Included Study

**FIGURE 1:**
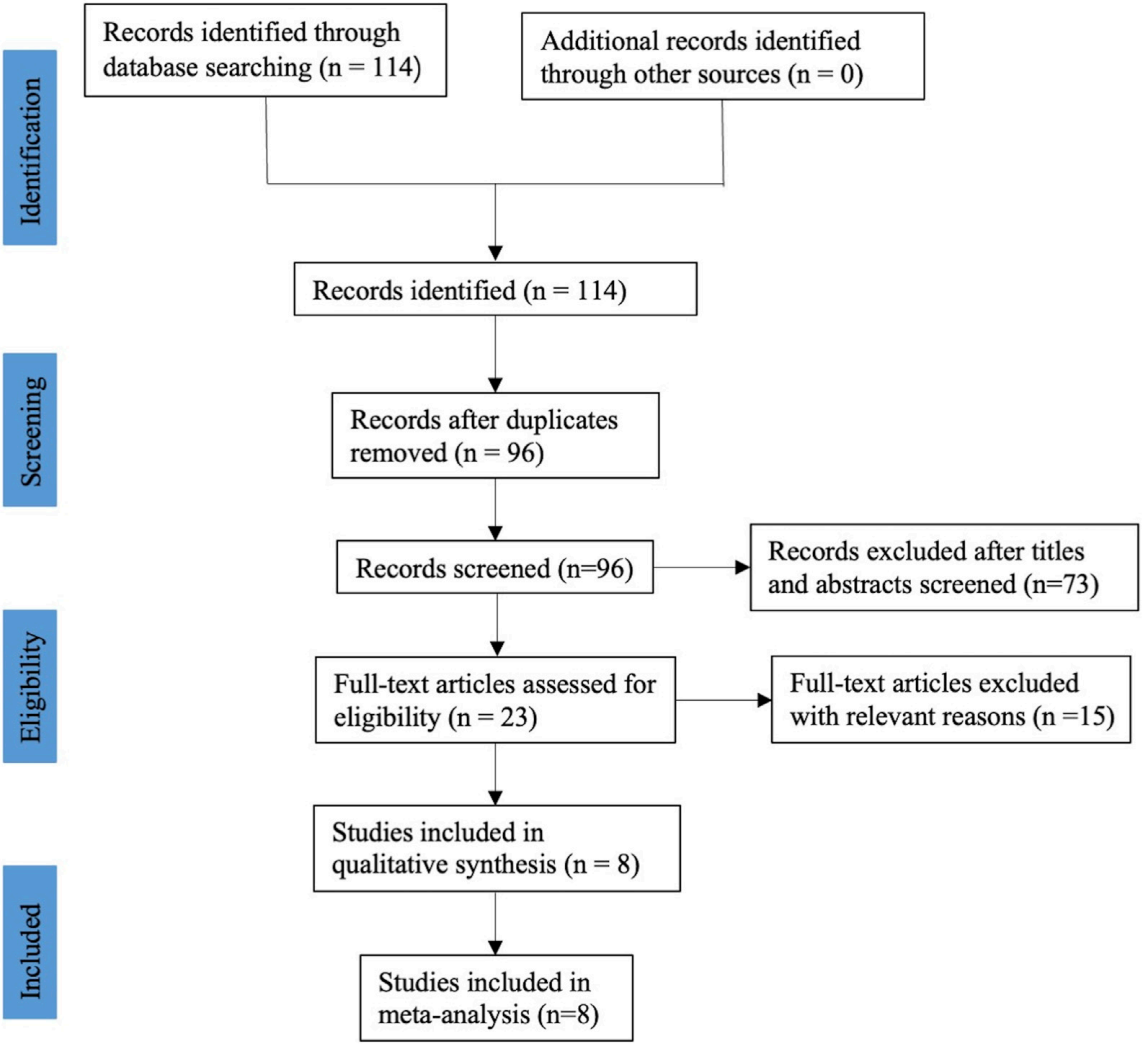
Flow chart of study selection.

After a systematic literature search, a total of one hundred and fourteen relevant publications were retrieved. After excluded duplicate records, 96 studies were screened using their titles and abstracts, and 73 of them were removed. Through reading the full text, fifteen records were excluded because of irrelevant to our topics, no relevant data and ongoing clinical research that has not yet been published. Finally, eight RCTs (Wu et al., 2017; Malahias et al., 2018; Raeissadat et al., 2018; Senna et al., 2019; Shen et al., 2019; Eltabl et al., 2020; Hashim et al., 2020; Chen et al., 2021b), including 436 participants, met the selection criteria and were included in this meta-analysis. The flow diagram involved in the current study is shown in Figure 1.

### Study Characteristics

The main characteristics and preparations of PRP injection among the eight included RCTs are shown in Tables 1, 2. In these enrolled studies, three studies were conducted in Egypt (Senna et al., 2019; Eltabl et al., 2020; Hashim et al., 2020), and three studies in China (Wu et al., 2017; Shen et al., 2019; Chen et al., 2021b), each one in Iran (Raeissadat et al., 2018) and Greece (Malahias et al., 2018). In these included studies, CTS patients were treated with local autologous PRP injection in the experimental group and different types of conservative treatment in the control group. The grades of included CTS patients ranged from mild to severe. Among control groups of all enrolled studies, two studies were treated with methylprednisolone acetate (Senna et al., 2019; Hashim et al., 2020), two studies with wrist splint (Wu et al., 2017; Raeissadat et al., 2018), two studies with 0.9% normal saline (Malahias et al., 2018; Chen et al., 2021b), each one with 5% dextrose (Shen et al., 2019), medical treatment and hand support (Eltabl et al., 2020). All included studies excluded patients with hematologic disorders such as thrombocytopenia, platelet dysfunction, and coagulation disorders or patients on recent treatment with NSAIDs, antiplatelets, and anticoagulants in order to reduce the incidence of injection-related adverse events. Six studies conducted PRP injection under ultrasound guidance (Wu et al., 2017; Malahias et al., 2018; Senna et al., 2019; Shen et al., 2019; Eltabl et al., 2020; Chen et al., 2021b). All studies were conducted with ulnar in plane approach to inject superficial and deep to the median nerve. Two studies reported injections performed under the short-axis view technique (Shen et al., 2019; Chen et al., 2021b). The level of injection was distal to the carpal joint in two studies (Raeissadat et al., 2018; Hashim et al., 2020) and proximal to the carpal tunnel in the remaining studies. Four studies reported injection needle sizes were 25 gauge (Wu et al., 2017; Raeissadat et al., 2018; Senna et al., 2019; Hashim et al., 2020). In only one study, local PRP injection complications occurred in four cases of itching, one case of finger pain, and one case of burning sensation (Raeissadat et al., 2018). A single injection of PRP was performed in all enrolled studies. PRP used in all included studies was derived from autologous peripheral blood of patients. The procedures for preparing PRP were described in detail in all included studies. The local injection dose of PRP ranged from 1 to 3.5 ml in enrolled studies. Two studies reported the type of PRP was leukocyte-rich (Wu et al., 2017; Shen et al., 2019), and two studies were leukocyte-poor (Raeissadat et al., 2018; Chen et al., 2021b). Only one study reported the concentration of platelets in PRP (Hashim et al., 2020). The published years of enrolled RCTs were between 2017 and 2021.

**TABLE 1:** The main characteristics of the included studies.

Study (Year)	Country	No. of patients		Male/Female	Age (Year)		Duration (Month)		CTS grading	Treatment in control group	Outcome measures	Follow-up
		PRP	Control		PRP	Control	PRP	Control				
Hashim et al., 2020	Egypt	20	20	5/35	48.8 ± 7.45	49.15 ± 6.06	24.1 ± 7.05	23.3 ± 7.26	Mild to moderate	1 ml methylprednisolone acetate	VAS, BCTQ, DML, SPL	3 months
Senna et al., 2019	Egypt	43	42	14/71	38.3 ± 6.4	40.7 ± 9.4	NR	NR	Mild to moderate	1 ml methyl prednisolone acetate	VAS, BCTQ, DML, SPL, SNCV, CSA	3 months
Shen et al., 2019	China	26	26	5/47	56.8 (31–73)	58.5 (31–77)	58.3 (3–360)	37.5 (3–120)	Moderate	3 ml 5% dextrose	BCTQ, SNCV, DML, CSA	6 months
Raeissadat et al., 2018	Iran	21	20	0/41	51.20 ± 9.82	47.23 ± 7.11	13.74 ± 11.5	14.13 ± 8.55	Mild to moderate	Wrist splint	VAS, BCTQ, SPL	7 weeks
Wu et al., 2017	China	30	30	8/52	57.87 ± 8.27	54.27 ± 7.34	34.43 ± 31.05	30.70 ± 33.03	Mild to moderate	Wrist splint	VAS, BCTQ, SNCV, DML, CSA	6 months
Eltabl et al., 2020	Egypt	30	30	24/36	37.93 ± 7.40	39.8 ± 7.39	NR	NR	Mild to moderate	Medical treatment and hand support	VAS, BCTQ, SPL, DML	6 months
Malahias et al., 2018	Greece	26	24	NR	57.17 ± 16.14	60.46 ± 14.39	>3	>3	Mild to moderate	2 ml 0.9% normal saline	CSA	12 weeks
Chen et al., 2021a	China	24	24	3/21	53 (31–74)	53 (31–74)	35.3 (3–120)	36.2 (1–120)	Moderate to severe	3.5 ml 0.9% normal saline	BCTQ, SNCV, DML, CSA	12 months

PRP; Platelet-Rich Plasma, CTS; carpal tunnel syndrome, NR; not reported.

**TABLE 2:** The characteristics of Platelet-rich plasma injection.

Study (Year)	Activation	Kit	Volume (ml)	Centrifuge time	Leukocyte classification	Injection way	Injection approach	Injection level	Needle size (gauge)
Hashim et al., 2020	Sodium citrate	NR	1	1,600 rpm (8 min)	NR	NR	Ulnar lateral	Distal carpal	25
Senna et al., 2019	Calcium chloride	Special PRP Kit (GD Medical Pharma)	2	3,000 rpm (3 min) then 4,000 rpm (15 min)	NR	Ultrasound-guided injection	Ulnar lateral	Proximal carpal	25
Shen et al., 2019	Sodium citrate and autologous thrombin	Regent Kit-THT-1 (RegenLab SA, Mont-sur-Lausanne, Switzerland)	3	3,400 rpm (15 min)	leukocyte-rich	Ultrasound-guided injection	Ulnar lateral	Proximal carpal	NA
Raeissadat et al., 2018	Anticoagulant citrate dextrose solution A	Rooyagen Kit (made by Arya Mabna Tashkis Corporation, RN:312,569)	1	1,600 rpm (12 min) then 3,500 rpm (7 min)	leukocyte-poor	NR	Ulnar lateral	Distal carpal	25
Wu et al., 2017	Sodium citrate and autologous thrombin	Regent Kit-THT-1 (RegenLab SA, Mont-sur-Lausanne, Switzerland)	3	3,400 rpm (15 min)	leukocyte-rich	Ultrasound-guided injection	Ulnar lateral	Proximal carpal	25
Eltabl et al., 2020	Sodium citrate	NR	1–2	3,500 rpm (9 min)	NR	Ultrasound-guided injection	Ulnar lateral	Proximal carpal	NA
Malahias et al., 2018	NR	NR	2	NR	NR	Ultrasound-guided injection	Ulnar lateral	Proximal carpal	NA
Chen et al., 2021b	Anticoagulant citrate dextrose solution-A	NR	3.5	500–1,200 g (8 min)	leukocyte-poor	Ultrasound-guided injection	Ulnar lateral	Proximal carpal	NA

Rpm; rotation per minutes, NR; not report.

### Quality Assessment of Individual Trials

**FIGURE 2:**
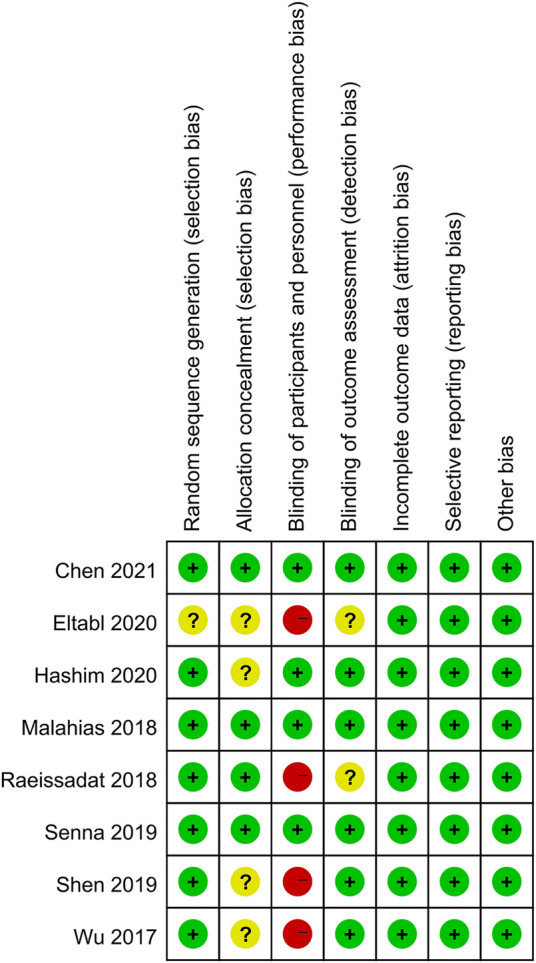
Risk of bias graph: low risk of bias in green; unclear risk of bias in yellow; high risk of bias in red.

**FIGURE 3:**
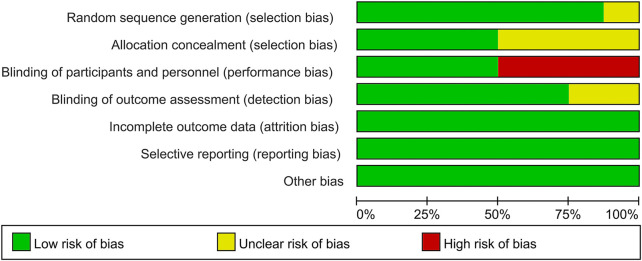
Risk of bias summary: each risk of bias item is presented as the percentage across all the included studies, which indicates the proportion of different levels of risk of bias for each item.

Among these enrolled studies, seven studies were performed with adequate random sequence generation (Wu et al., 2017; Malahias et al., 2018; Raeissadat et al., 2018; Senna et al., 2019; Shen et al., 2019; Hashim et al., 2020; Chen et al., 2021b). In addition, four studies were conducted with allocation concealment (Malahias et al., 2018; Raeissadat et al., 2018; Senna et al., 2019; Chen et al., 2021b), and the remaining four studies were not reported and determined to be unclear. Blinding of participants and personnel was reported in four included studies (Malahias et al., 2018; Senna et al., 2019; Hashim et al., 2020; Chen et al., 2021b). The remains cannot be blinded because of the treatment method. Blinding of outcome assessment was reported in six included studies (Wu et al., 2017; Malahias et al., 2018; Senna et al., 2019; Shen et al., 2019; Hashim et al., 2020; Chen et al., 2021b). In addition, the outcome reports and data of all studies are complete. And we did not identify other obvious sources of bias in the trials. Figures 2, 3 summarized the methodological quality of all enrolled studies.

### Meta-Analysis of Short-Term Efficacy

**FIGURE 4:**
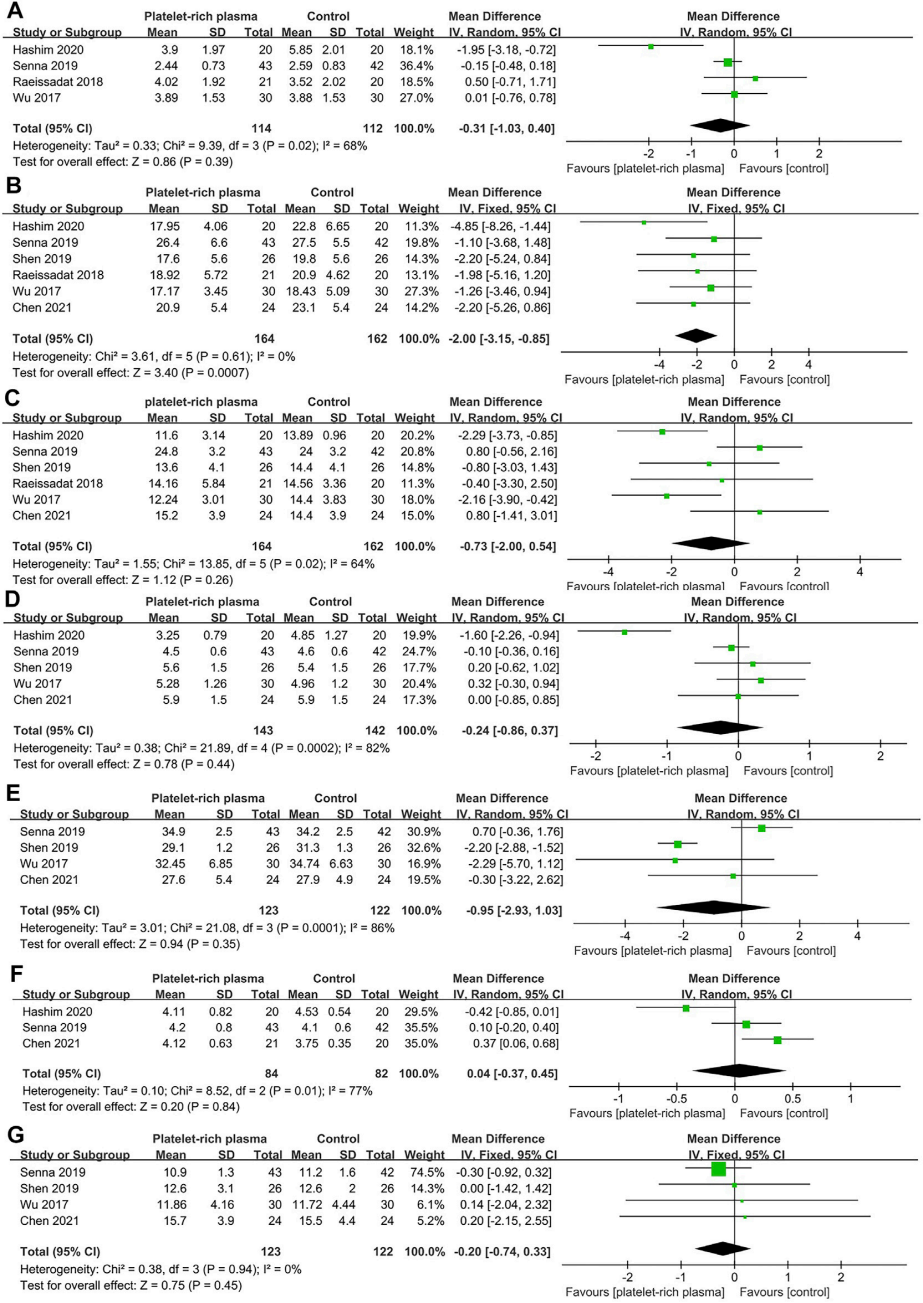
Forest plot showing short-term efficacy of local platelet-rich plasma injection versus other conservative treatments. **(A)** Visual analogue scale. **(B)** Symptom severity scale. **(C)** Functional status scale. **(D)** Distal motor latency. **(E)** Sensory nerve conduction velocity. **(F)** Sensory peak latency. **(G)** Cross sectional area.

A total of six studies reported data on short-term efficacy after no-surgical treatment (164 and 162 patients in the PRP and control groups, respectively). The results of pooled analyses were shown in Figure 4. The results showed that the SSS of local PRP injection group were significantly lower than that of control group (MD = −2.00; 95% CI, −3.15 to −0.85; *p* < 0.001), However, there were no significant differences between two groups in VAS score (MD = −0.31; 95% CI, −1.03 to 0.40; *p* = 0.39), FSS (MD = -0.73; 95% CI, −2.00 to 0.54; *p* = 0.26), DML(MD = −0.24; 95% CI, −0.86 to 0.37; *p* = 0.44), SNCV(MD = −0.95; 95% CI, −2.93 to 1.03; *p* = 0.35), SPL(MD = −0.04; 95% CI, -0.37 to 0.45; *p* = 0.84), CSA(MD = -0.20; 95% CI, -0.74 to 0.33; *p* = 0.45).

### Meta-Analysis of Mid-term Efficacy

**FIGURE 5:**
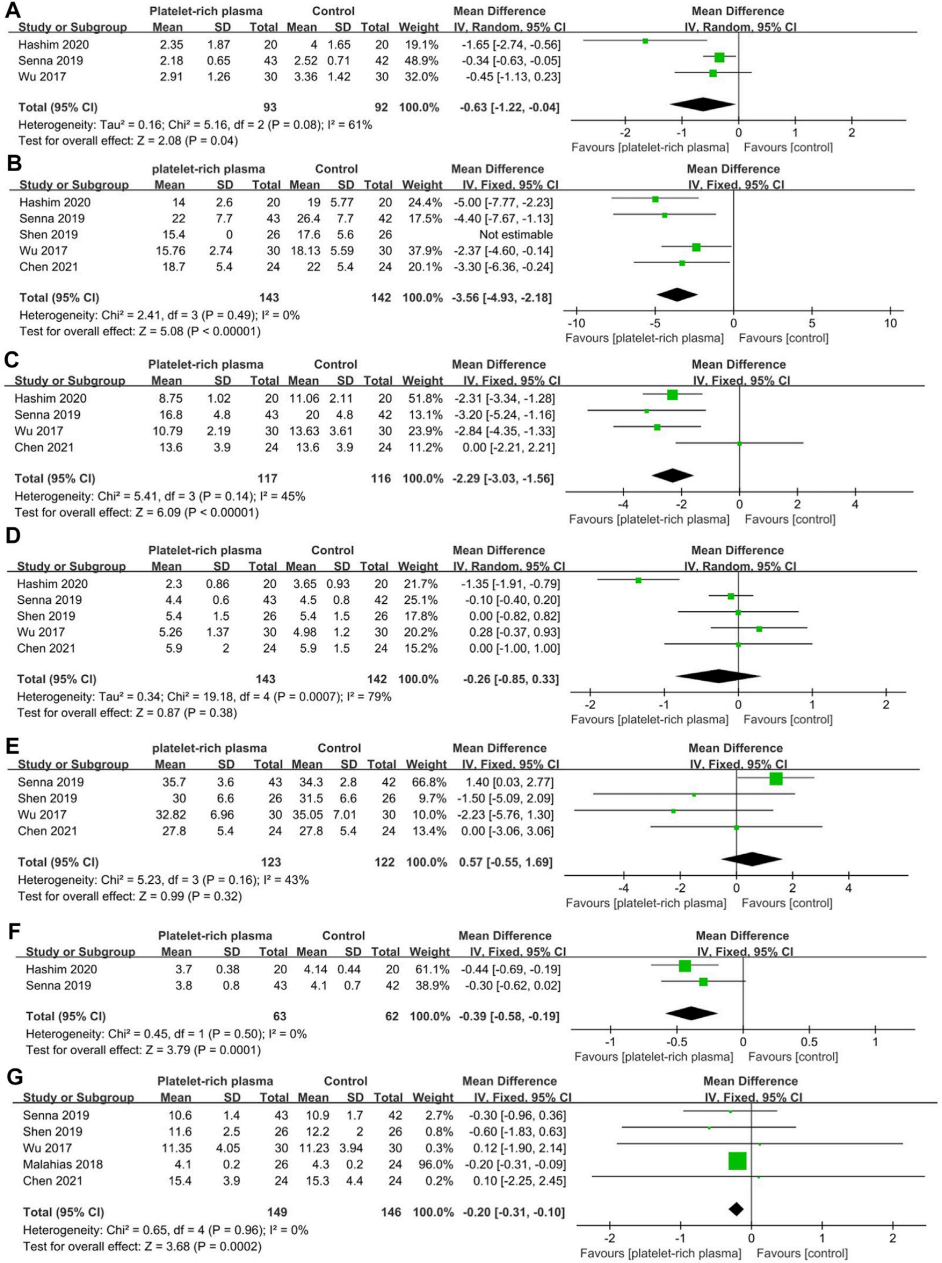
Forest plot showing mid-term efficacy of local platelet-rich plasma injection versus other conservative treatments. **(A)** Visual analogue scale. **(B)** Symptom severity scale. **(C)** Functional status scale. **(D)** Distal motor latency. **(E)** Sensory nerve conduction velocity. **(F)** Sensory peak latency. **(G)** Cross sectional area.

A total of six studies reported data on mid-term efficacy after no-surgical treatment (169 and 166 patients in the PRP and control groups, respectively). The results of pooled analyses are shown in Figure 5. The results showed that the SSS of the local PRP injection group was significantly lower than that of the control group (MD = −0.63; 95% CI, −1.22 to −0.04; *p* = 0.04). Besides, the SSS and FSS of the PRP group were significantly lower than that of the control group (MD = −3.56; 95% CI, −4.93 to −2.18; *p* < 0.001, MD = −2.29; 95% CI, −3.03 to −1.56; *p* < 0.001). The CSA was significantly reduced in the PRP group than that of the control group (MD = −0.20; 95% CI, −0.31 to −0.10; *p* < 0.001). In terms of electrodiagnostic examination parameters, PRP group was significantly reduced in SPL (MD = −0.39; 95% CI, −0.58 to −0.19; *p* < 0.001). In addition, there were no significant differences between two groups in DML (MD = −0.26; 95% CI, −0.85 to 0.33; *p* = 0.38) and SNCV (MD = 0.57; 95% CI, −0.55 to 1.69; *p* = 0.32).

### Meta-Analysis of Mid-Long-Term Efficacy

**FIGURE 6:**
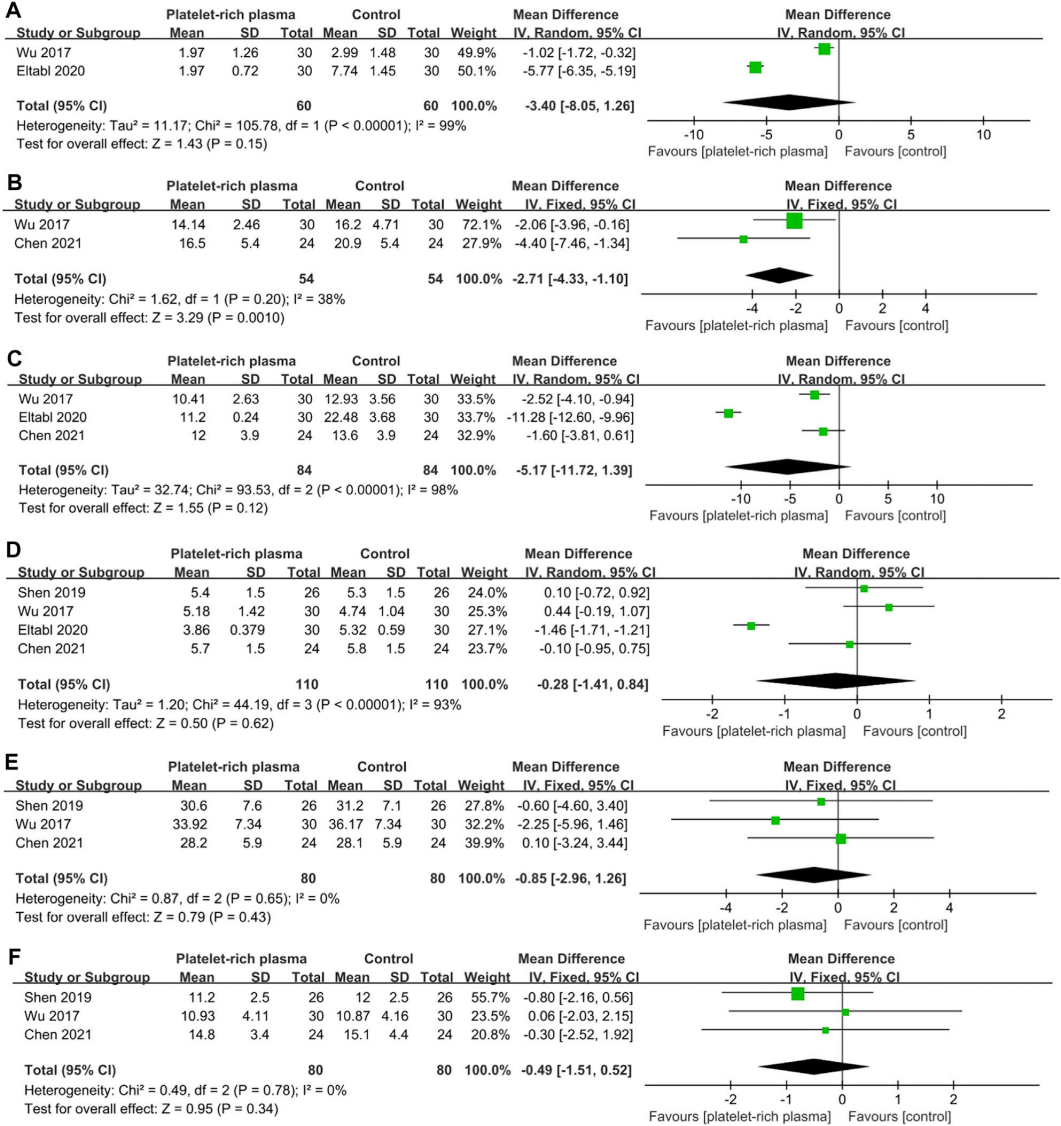
Forest plot showing mid-long-term efficacy of local platelet-rich plasma injection versus other conservative treatments. **(A)** Visual analogue scale. **(B)** Symptom severity scale. **(C)** Functional status scale. **(D)** Distal motor latency. **(E)** Sensory nerve conduction velocity. **(F)** Cross sectional area.

A total of four studies reported data on mid-long-term efficacy after no-surgical treatment (110 and 110 patients in the PRP and control groups, respectively). The results of pooled analyses were shown in Figure 6. The results showed that the SSS of local PRP injection group were significantly lower than that of control group (MD = −2.71; 95% CI, −4.33 to −1.10; *p* = 0.001), However, there were no significant differences between two groups in VAS score (MD = −3.40; 95% CI, −8.05 to 1.26; *p* = 0.15), FSS (MD = −5.17; 95% CI, −11.72 to 1.39; *p* = 0.12), DML(MD = −0.28; 95% CI, −1.41 to 0.84; *p* = 0.62), SNCV (MD = −0.85; 95% CI, −2.96 to 1.26; *p* = 0.43) , CSA(MD = −0.49; 95% CI, −1.51 to 0.52; *p* = 0.34).

### Sensitivity Analysis

A sensitivity analysis was performed by individually removing each study to determine whether the pooled results changed. The pooled results at different time were stable.

## Discussion

In our study, we conducted a systematic review and meta-analysis to compare the clinical efficacy of PRP injection and other conservative treatments in CTS patients. The results showed that local injection of PRP is more effective than other conservative treatments in decreasing SSS score in the short-term follow-up. In the mid-term follow-up, the local PRP injection was better than other conservative treatments in terms of VAS, SSS, FSS, SPL, and CSA. In the mid-long-term follow-up, the SSS score of local injection of PRP was higher than that of control group.

The symptoms of CTS would become severe without treatment over time. Long-term compression and ischemia of the MN in the carpal tunnel may cause permanent nerve damage and aseptic inflammation. Traditional conservative treatments, including oral medications, musculoskeletal manipulation, and wrist splints, can alleviate local pain, but their repair effects on nerve function are limited (Padua et al., 2016). A local steroid injection can effectively reduce the expression of acute inflammatory factors such as CRP and IL-6 to achieve anti-inflammatory and pain relief effects (Peerbooms et al., 2019; Hashim et al., 2020). However, the application of steroids is not related to the activation of repair-related signaling pathways and will increase the risk of local soft tissue rupture and neurotoxicity (Nepple and Matava, 2009; Kim and Park, 2014). At present, many studies demonstrated that PRP had a positive effect on peripheral nerve regeneration, anti-inflammatory and regulating angiogenesis (Sánchez et al., 2012). Zheng et al. proved that PRP at a concentration of 2.5%–20% could significantly stimulate the proliferation and migration of Schwann cells *in vitro* and promoted the secretion of nerve growth factor and glial cell line-derived neurotrophic factor (Zheng et al., 2016). Farrag et al. proved that in the rat facial nerve axotomy model, the addition of PRP in the process of nerve suture could significantly increase the thickness of myelin sheath and the number of axons, and the speed of nerve transmission is significantly increased (Farrag et al., 2007). Therefore, injecting PRP around the diseased nerve tissue might be a promising bioremediation treatment.

At present, the biological effect mechanism of PRP is mainly through the activation of platelet concentrates on releasing a large number of cytokines, such as platelet-derived growth factor, transforming growth factor-β, insulin-like growth factor and, vascular endothelial growth factor, which have powerful effects on cell proliferation and tissue repair (Picard et al., 2015). All these cytokines can not only directly repair damaged MN, but also increase the production of α-2 collagen and type III collagen in the flexor support belt cells to reduce the pressure in the carpal tunnel (Allampallam et al., 2000)

Our meta-analysis results showed that the SSS score in the PRP group was significantly lower than that in the control group in the short-term follow-up. VAS, SSS, FSS, SPL, and CSA in the PRP group all improved significantly during the mid-term follow-up. In our opinion, we think that PRP have a certain delay effect in exerting its functions, and it takes some time to reach its maximum clinical effect. A similar phenomenon also appeared when using PRP to treat plantar fasciitis and external humeral epicondylitis (Merolla et al., 2017; Fei et al., 2021). In addition, among the studies that reported short-term efficacy, four studies conducted local PRP injections under ultrasound guidance (Wu et al., 2017; Senna et al., 2019; Shen et al., 2019; Chen et al., 2021b). During the injection process, the hydrodissection effect around the nerve sheath helps to reduce the symptoms of CTS, which refers to stripping the adhesion and compression of the related connective tissue and the flexor retinaculum around the MN to reduce the ischemic damage caused to it (Cass, 2016). A single-blind RCT showed that ultrasound-guided injection is more effective than blind injection in alleviating the symptoms of CTS in the early stage (Ustün et al., 2013). Additionally, local injection guided by ultrasound can also avoid iatrogenic MN injury and reduce complications. All these results confirmed that the close equivalent effect of PRP versus other conservative treatments in the short-term efficacy and the mid-term effects of PRP significantly improved.

During the mid-long-term follow-up, the SSS score of the PRP group was significantly lower than that of the control group. However, no significant differences were found in other outcome indicators, which is consistent with the results of previous study (Uzun et al., 2017). They believe that this temporary efficacy is attributable to the dose and frequency of PRP injections. After PRP is injected into the patient, the local repairability may decrease as platelets and growth factors are degraded. Through timely re-injection, a higher concentration of growth factors in the damaged tissues of the patient’s carpal tunnel can be maintained to achieve long-term and effective promotion of CTS patients to improve clinical symptoms and wrist function. Moreover, no correlations between the improvement of symptoms and the results of the electrodiagnostic examinations were found during follow-up periods. This inconsistency is expected because the conventional electrodiagnostic examination mainly evaluates large myelinated fibers rather than small sensory fibers, and these feeling fibers may be associated with many CTS symptoms (Soyupek et al., 2012).

The studies included in our meta-analysis included differences in the preparation methods, platelet concentration, and injection volume. There is no consensus on the preparation process and the optimal injection concentration and PRP volume for CTS patients’ treatment. The time interval between PRP preparation and injection was not explicitly described in all studies, and to our knowledge, the time from extraction to preparation to injection of PRP is relatively fast and the impact on outcome indicators is negligible. However, only one study reported platelet concentration (Hashim et al., 2020). The previous study showed that the platelet concentration of therapeutic PRP should be 4–6 times that of whole blood, cause low concentration might be ineffective in the healing process (Crane and Everts, 2008). Different types of activations in preparation of PRP could affect the release of growth factors (Harrison et al., 2011). Four studies used a 25-gauge needle for injection, the remaining studies did not specify. Local PRP injection with different needle sizes and calibres has been proved to not influence platelet functionality, so smaller-sized needles should be chosen to minimize pain during the injection (Bausset et al., 2014). In addition, the injection volume of PRP in the enrolled studies ranges from 1 to 3.5 ml. The biological effects of different injection volumes are different. The hydrodissection effect and repairability can be more noticeable when the large injection volume. PRP of rich and poor leukocytes also differ in their efficacy. However, due to limited published studies, it is necessary to conduct further research on the optimal preparation and volume of local injection of PRP in the future.

The SSS score of the PRP group was significantly lower than that of the control group, and the FSS score was significantly lower than that of the control group during the mid-term follow-up. It can be concluded that local injection of PRP in the treatment of CTS effectively relieves symptoms and improves wrist joint function, and the mid-term efficacy is the most obvious. Therefore, local PRP injection may be a better and more promising treatment compared with other conservative treatments.

As for complications after local injection of PRP, only two studies reported injection-related adverse events. The study by Raeissadat et al. showed four cases of itching, one case of finger pain and one case of burning sensation after local injection (Raeissadat et al., 2018). Senna et al. reported an increase in pain sensation in the first 48 h following injection, which was relieved by the administration of paracetamol and local ice application (Senna et al., 2019). Due to the widespread use of ultrasound guidance, serious complications such as vascular and nerve injury caused by local injection are rare. And all the studies have used the ulnar lateral approach. According to a Bayesian network meta-analysis, ultrasound-guided ulnar injection is the most effective injection method for the treatment of carpal tunnel syndrome (Chen et al., 2015). PRP prepared from autologous blood avoids immune reactions and its bioprosthetic effect also reduces the risk of tendon rupture due to local steroid injection. Therefore, local PRP injection in CTS patients is a relatively safe therapy.

There are several limitations to our study. Firstly, our study is only a summary analysis of the mid-long-term efficacy of local injection of PRP, and the follow-up time is relatively short. Secondly, this study reported more outcome indicators from short-term efficacy, mid-term efficacy, and mid-long-term efficacy, including functional and symptom scores, electrodiagnostic examinations, and CSA of MN in CTS patients. Thirdly, although all enrolled studies are RCTs, the sample size of each study is small. Despite the limitations, the current meta-analysis found that local injection of PRP is a more effective treatment for CTS.

## Conclusion

This systematic review indicates that local PRP injection is more effective than other conservative treatments in terms of mid-term efficacy in relieving pain, improving wrist function and symptoms, reducing MN swelling, and partially improving electrophysiological indicators. In terms of short-term and mid-long-term efficacy, PRP can only improve wrist symptoms compared with other conservative treatments, and there is no significant difference in other aspects. Therefore, local autologous PRP injection is a safe and effective treatment in CTS patients compared with other conservative treatments. However, more research is needed in the future to explore the long-term efficacy of local PRP injection in the treatment of CTS and the unified process of PRP preparation and optimal concentration and dose.

## Data Availability

The original contributions presented in the study are included in the article/Supplementary Material, further inquiries can be directed to the corresponding author.
